# Post‐ROSC ST‐Segment Elevation: A Diagnostic Pitfall in Cardiac Arrest Survivors

**DOI:** 10.1111/anec.70187

**Published:** 2026-03-31

**Authors:** Oğuz Kaan Kaya

**Affiliations:** ^1^ Department of Cardiology Antalya Training and Research Hospital Antalya Türkiye

Dear Editor,

ST‐segment elevation detected immediately after return of spontaneous circulation (ROSC) in survivors of cardiac arrest frequently raises concern for acute coronary occlusion and often leads to urgent coronary angiography. However, accumulating evidence suggests that transient ST‐segment elevation in this setting does not always represent an acute culprit coronary lesion.

Several mechanisms may contribute to ST‐segment elevation following ROSC. Global myocardial ischemia during cardiac arrest and subsequent reperfusion injury can produce transient repolarization abnormalities. In addition, severe metabolic disturbances, catecholamine surge, and myocardial stunning may alter myocardial electrical activity and mimic electrocardiographic patterns typically associated with acute coronary occlusion (Lemkes et al. 2019; Spaulding et al. 1997). These mechanisms may result in transient ST‐segment elevation despite the absence of obstructive coronary artery disease.

Importantly, studies evaluating coronary angiography after cardiac arrest have demonstrated that a substantial proportion of patients without ST‐segment elevation do not have an acute culprit lesion, highlighting the complexity of electrocardiographic interpretation in the post‐resuscitation phase (Lemkes et al. 2019). Conversely, transient ST‐segment elevation immediately after ROSC may occasionally reflect global ischemia or reperfusion‐related electrical instability rather than true ST‐elevation myocardial infarction.

While early coronary angiography remains essential in many patients with suspected acute coronary syndrome, reliance solely on ST‐segment elevation immediately after ROSC may occasionally lead to diagnostic uncertainty. Careful integration of clinical findings, hemodynamic status, echocardiographic assessment, and serial electrocardiograms may help distinguish transient post‐resuscitation ECG changes from true coronary occlusion (Ibanez et al. 2018).

Greater awareness of this diagnostic challenge may improve interpretation of electrocardiographic findings after cardiac arrest and help guide more individualized decision‐making regarding coronary angiography in cardiac arrest survivors.

## Conflicts of Interest

The author declares no conflicts of interest.

## Data Availability

The data that support the findings of this study are available on request from the corresponding author. The data are not publicly available due to privacy or ethical restrictions.
